# Use of Natural Products in Asthma Treatment

**DOI:** 10.1155/2020/1021258

**Published:** 2020-02-13

**Authors:** Lucas Amaral-Machado, Wógenes N. Oliveira, Susiane S. Moreira-Oliveira, Daniel T. Pereira, Éverton N. Alencar, Nicolas Tsapis, Eryvaldo Sócrates T. Egito

**Affiliations:** ^1^Graduate Program in Health Sciences, Dispersed System Laboratory (LaSid), Pharmacy Department, Federal University of Rio Grande do Norte (UFRN), Av. General Gustavo de Cordeiro-SN-Petrópolis, Natal 59012-570, Brazil; ^2^Institut Galien Paris-Sud, CNRS, Univ. Paris-Sud, Université Paris-Saclay, 92296 Châtenay-Malabry, France; ^3^Graduate Program in Pharmaceutical Nanotechnology, LaSid, UFRN, Av. General Gustavo de Cordeiro-SN-Petropolis, Natal 59012-570, Brazil

## Abstract

Asthma, a disease classified as a chronic inflammatory disorder induced by airway inflammation, is triggered by a genetic predisposition or antigen sensitization. Drugs currently used as therapies present disadvantages such as high cost and side effects, which compromise the treatment compliance. Alternatively, traditional medicine has reported the use of natural products as alternative or complementary treatment. The aim of this review was to summarize the knowledge reported in the literature about the use of natural products for asthma treatment. The search strategy included scientific studies published between January 2006 and December 2017, using the keywords “asthma,” “treatment,” and “natural products.” The inclusion criteria were as follows: (i) studies that aimed at elucidating the antiasthmatic activity of natural-based compounds or extracts using laboratory experiments (*in vitro* and/or *in vivo)*; and (ii) studies that suggested the use of natural products in asthma treatment by elucidation of its chemical composition. Studies that (i) did not report experimental data and (ii) manuscripts in languages other than English were excluded. Based on the findings from the literature search, aspects related to asthma physiopathology, epidemiology, and conventional treatment were discussed. Then, several studies reporting the effectiveness of natural products in the asthma treatment were presented, highlighting plants as the main source. Moreover, natural products from animals and microorganisms were also discussed and their high potential in the antiasthmatic therapy was emphasized. This review highlighted the importance of natural products as an alternative and/or complementary treatment source for asthma treatment, since they present reduced side effects and comparable effectiveness as the drugs currently used on treatment protocols.

## 1. Introduction

### 1.1. Physiopathology of Asthma

Asthma can be defined as a chronic inflammatory disorder that affects the lower airways, promoting an increase of bronchial reactivity, hypersensitivity, and a decrease in the airflow [1]. Furthermore, due to a complex interaction between the genetic predisposition and environmental factors, besides multiple related phenotypes, this disease may be considered as a heterogeneous disorder [2].

**Figure 1:**
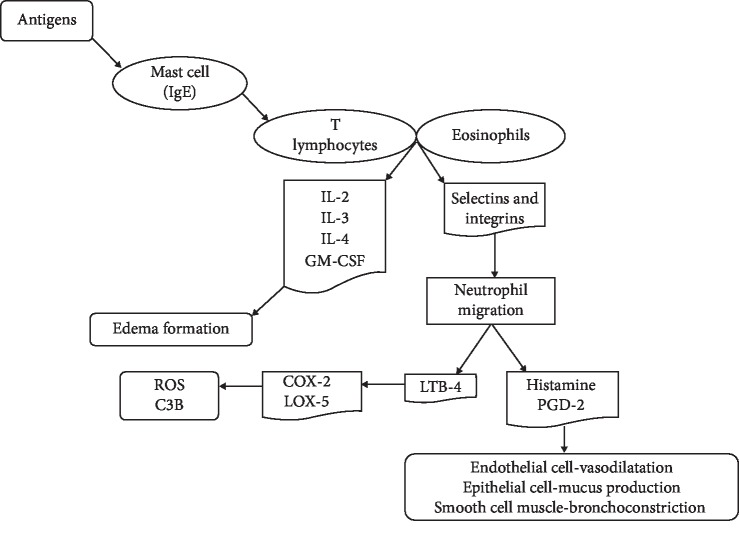
Scheme of the immune response induced by allergen or antigen stimulation or the early stages of asthma. GM-CSF: granulocyte-macrophages colony-stimulating factor; IL: interleukin; C3b: opsonin; LOX-5: lipoxygenase type 5; ROS: reactive oxygen species; COX-2: cyclooxygenase type 2; LTB_4_: leukotriene type B; PGD_2_: prostaglandin type D (adapted from Bradding et al. [6].

Sensitization by dust, pollen, and food represents the main environmental factors involved in the asthma physiopathology [1]. These antigens are recognized by the mast cells coated by IgE antibodies (Figure 1) and induce the release of proinflammatory cytokines, such as tumor necrosis factor-*α* (TNF-*α*), interleukins IL-2, IL-3, IL-4, IL-5, GM-CSF, prostaglandins, histamine, and leukotrienes [3, 4], by T lymphocytes and eosinophils. This degranulation process promotes an increase in the vascular permeability, leading to exudate and edema formation. This process is followed by leukocyte migration to the tissue affected by the inflammatory process through chemotaxis mediated by selectins and integrins [3, 6]. Subsequently, the neutrophil migration to the inflammatory site and the release of leukotrienes LTB_4_ induce the activation of type 2 cyclooxygenase (COX-2) and type 5 lipoxygenase (LOX-5), enhancing the expression of the C3b opsonin that produces reactive oxygen species (ROS) and thus promoting cell oxidative stress and pulmonary tissue injury [3, 7].

Other mechanisms involved in asthma physiopathology are the inhalation of drugs, as well as respiratory viruses [8], which promote an immune response mediated by IgG antibodies. This process promotes an increase of the inflammatory cells influx, releasing inflammatory mediators responsible for the damage process [9].

Based on the factors and mechanisms presented above, asthma symptoms can be observed at different levels according to etiology and severity of clinical aspects, which define their classification [10]. The asthma severity is subdivided into (i) mild/low, also defined as intermittent/persistent, when the symptoms appear more than twice a week and their exacerbations can affect the daily activities of the patient; (ii) moderate, in which the daily symptom occurrence and their exacerbations affect the patient activities, requiring the use of short-acting *β*2-adrenergic drugs; or (iii) severe asthma, in which the patient presents persistent symptoms, physical activity limitations, and frequent exacerbations [10]. Based on this classification, it is estimated that 60% of the asthma cases are intermittent or persistent, 25% to 30% are moderate, and the severe cases account for only 10% of the total. However, it is important to highlight that although the proportion of severe asthmatics represents the minority of the cases, they are responsible for high mortality and high hospitalization costs [11], evidencing the high need of efficient treatments for this disease.

### 1.2. Asthma Epidemiology

According to the World Health Organization, asthma affects about 300 million of individuals across the world, regardless of the country development degrees [12]. In the United Kingdom, asthma affects approximately 5.2 million of individuals and is responsible for 60.000 hospital admissions per year [13], while in Brazil the annual incidence of hospital admissions due to asthma is around 173.442 patients, representing 12% of the total admissions for respiratory diseases in 2012 [14].

Furthermore, studies have demonstrated that asthma incidence and prevalence rates in different countries are not age related. In the United States of America, Albania, and Indonesia, the asthma prevalence is lower for children (around 8.4%, 2.1%, and 4.1%, respectively) when compared to adults [15]. On the other hand, in countries such as the United Kingdom and Costa Rica, children aged between 6 and 7 years represent approximately 32% of the asthma prevalence [16]. Additionally, the incidence or prevalence can be directly influenced by the socioeconomic characteristics of specific areas, as demonstrated by the analyses of the annual variation in the prevalence of asthma in which it was possible to observe, in Spain, that the asthma prevalence had an annual increase of 0.44% regardless of the age range studied. However, when these same data analyses were observed individually in Spain regions, the annual variation presented a different scenario, showing an increase or decrease according to the developmental degree of each region [17, 18]. Similar data were observed by Pearce et al. [19] and Schaneberg et al. [20], who demonstrated the influence of socioeconomic aspects on asthma. The studies showed a prevalent increase of asthma cases in metropolitan areas, fact attributed to the population growth with consequent exposure to the environmental factors and shortened access to asthma therapy, due to the high cost of the available medicines [19, 20]. Such phenomena directly interfere on the treatment compliance [21–23], evidencing the importance and the need of strategies that facilitate the access to the medicines for asthma therapy.

Studies that evaluate the importance of inclusion of antiasthmatic therapy on public health policy programs have demonstrated that asthma control can be achieved through a variety of approaches, promoting a decrease in hospital admissions of 90%. Indeed, this was demonstrated by two studies performed in Brazilian cities, in which public health programs offered free medicines and psychological and pharmaceutical care to treat chronic diseases [24, 25]. Furthermore, Ponte et al. [24] and Holanda [25] also showed that the hospital admissions of children decreased from 44.7% to 6.4% one year after the inclusion of these patients in the same project. Thus, these data corroborate the importance of public health policies that contribute to the reduction of hospital outlay, increasing the population's life quality.

### 1.3. Asthma Treatment

The asthma treatment recommended by the Global Initiative for Asthma (GINA) consists, especially, on the reduction of symptoms in order to decrease the inflammatory process [26, 27]. However, since asthma presents a complex physiopathology associated with variable manifestations, the treatment can lead to different response levels. Thus, the evaluation of the clinical aspects associated with the treatment response is defined as the most adequate approach to achieve treatment success [28]. Asthma therapy strategies are based on pulmonary (the main administration route on asthma therapy), oral or intravenous administration of class *β*2 agonist drugs (salbutamol, levalbuterol, terbutaline, and epinephrine), anticholinergics (ipratropium), corticosteroids (beclomethasone di- or monopropionate, ciclesonide, flunisolide, fluticasone propionate, mometasone furoate, triamcinolone acetonide, hydrocortisone, dexamethasone, budesonide, prednisone, prednisolone, and methylprednisolone), and xanthine drugs. Among these, the *β*2 agonists are often the drugs of first choice [13, 27].

To optimize the treatment for each patient, the drug dosage is determined by the patient's respiratory characteristics, mainly his/her respiratory rate. Patients with increased respiratory rate, due to the airways narrowing, present a low dispersion of the inhaled drug through the respiratory tract [29]. In these cases or when there is an absence of response on the first two hours after treatment, hospitalization should be performed, and adrenaline could be used, subcutaneously or intravenously, since this is an indicative of mucosal edema formation, which can be decreased by the adrenaline bronchodilator effect [30].

Overall, patients that present asthma exacerbation should be initially treated with the association of different dosage of corticosteroids and short-acting *β*2 agonists by intranasal oxygen administration, allowing the stimulation of *β*2 receptors that result in bronchodilation due to the inhibition of cholinergic neurotransmission and, thus, inhibition of mast cells degranulation [10]. Additionally, corticosteroids by oral or inhaled route are used on uncontrolled persistent asthma patients due to their direct effect on the inflammation site [31]. Accordingly, they improve the pulmonary function and decrease the asthma episodes [32], reducing hospitalizations and mortality of asthmatic patients [31]. Furthermore, because their systemic use can induce side effects, corticosteroids, mainly prednisone and prednisolone, are more commonly used in patients with severe persistent asthma who are not stabilized by other drugs [31].

In addition, xanthine drugs such as theophylline can be also used on asthma treatment, since they are able to promote the suppression of monocyte activation with consequent inhibition on the TNF-*α* release. Further, they promote the inhibition of neutrophil activation and its degranulation, inhibiting the catalytic activity of phosphodiesterase 4 (PDE4), allowing a reduction in the inflammatory process [33].

Regardless of the wide variety and associations of antiasthmatic medicines and their ability to promote the asthma symptoms control and to reduce the asthma episodes and hospital admissions, the antiasthmatic drugs present several side effects, including nausea, headaches, and convulsions (xanthine class) [3, 30], cardiovascular effects (*β*-adrenergic receptors antagonists) [20], vomiting (PDE4 inhibitors drugs) [34–36], osteoporosis, myopathies, adrenal suppression, and metabolic disturbs, compromising the patients' growth (corticosteroids) [30, 35, 37, 38]. These side effects compromise the life quality of the patients and reduce significantly the treatment compliance.

Another important drawback from the conventional asthma treatment is its cost. In fact, the required amount of money for asthma treatments represents a significant expenditure for health public organizations. Such situation has become a financial issue even for developed countries. In Sweden, for example, the cost of medicines for asthma treatment has increased since the 1990s and, in 2006, and it was responsible for 11.6% of the total healthcare expenditure. Furthermore, according to projections, an annual increase of 4% on the costs of asthma management is expected [22].

Additionally, studies revealed that in Europe and in the United States of America, the sum of the direct and indirect estimated annual costs with asthma management is approximately €18 billion and US$13 billion, respectively. This high expenditure was associated with the high incidence of uncontrolled asthma patients, since they represent an expense up to 5-fold higher than the controlled asthma ones [39] or than patients with other chronic diseases, as demonstrated in the study performed by O'Neil and colleagues [40]. These authors revealed that asthma costs up to £4,217 per person, while type II diabetes, chronic obstructive pulmonary disease, and chronic kidney disease represented, together, a cost of £3,630 [40].

Therefore, considering the therapies currently available, their side effects, and their high cost, the development of new therapeutic approaches or complementary treatments to the current asthma therapy become an important and essential strategy. In this context, the use of natural products allows easy access to treatment to all socioeconomic classes [41, 42] and shows advantages such as low cost, biocompatibility, and reduced side effects, besides their wide biodiversity and renewability [43, 44]. In addition, natural products, supported by the literature findings on their complex matrix as a source of bioactive compounds, represent one of the main access forms to the basic healthcare in the traditional medicine [45]. Thus, the present review aimed at summarizing the main natural products reported in the literature that show antiasthma activity.

## 2. Natural Products as Alternative for Asthma Treatment

The use of natural products for the treatment of physiologic disorders, especially in association with other drugs, has been widely reported through ethnopharmacological studies as an important scientific tool for bioprospection exploration and discovery of new bioactive compounds from natural sources [46]. Despite the wide scientific progress regarding chemical and pharmaceutical technology on synthesizing new molecules, drugs from natural sources still contribute tremendously to the discovery and development of new medicines [47]. These studies are based, initially, on the traditional use of the natural products, which draws the attention of pharmaceutical companies due to their easy and economical use, allowing the companies to perform many studies that evaluate their therapeutic activities, their toxicity, and their safety [48].

Moreover, the use of natural products as complementary therapy represents an important alternative for the treatment of several diseases [49]. In the United States of America, the use of natural products, vitamins, and other dietary supplements as auxiliary treatments represent about 40% of the conventional therapies [50]. Among the diseases that natural products are used for, those of allergic and inflammatory character can be highlighted. In fact, according to the literature, the alternative medicine associates the use of these products with biochemical mechanisms involved in immunomodulation, which could contribute to the management of these diseases [51].

The use of plant-based products for asthma treatment has been reported by the traditional medicine for over 5000 years, since the use by the Chinese culture of the infusion of *Ephedra sinica*, which is as an immune system stimulator able to decrease asthma crises [20]. More recently, a study performed by Costa and colleagues [49] described the main natural sources for the treatment of asthma used by the Brazilian families from the Northeast Region of the country [49]. The study included beet, honey, onion, lemon, garlic, yarrow, and mint, demonstrating the wide variety of natural products used on asthma treatment in children [49]. Additionally, other natural-derived products have been widely cited in asthma treatment, such as natural oils from plants and animals, which can be obtained by different extraction process [52, 53].

Plant-derived natural oils represent the main natural products used on the complementary asthma therapy due to the presence of compounds such as phenylpropanoids and mono- and sesquiterpenes as the major bioactive compounds, which provide their anti-inflammatory, antifungal, antibacterial, and anesthetic properties [54–56]. Similarly, oils obtained from animal sources have been used. They are rich in a mixture of different saturated, mono and polyunsaturated fatty acids, as well as compounds from animal organs and secretions, which are responsible for the immune-modulatory action and regulation of the tissue oxidative capacity [57, 58]. The activity credited to the oils derived from plants and animals is related to the presence of those bioactive compounds, which can inhibit COX-2 and COX-5. Additionally, these compounds are able to modulate the immune cells function by reducing levels of IL-4, IL-5, and IL-13 cytokines, decreasing the activity and proliferation of NK cells and leading to an increase in the level of endogenous corticosteroids, contributing to the regulation of NF-*κ*B pathway, and reducing the mucus production and the inflammation in the lung tissues [59–61].

In this regard, Table 1 shows all products found in the studies included in this review after the inclusion criteria evaluation. Due to the wide variety of plant-derived products, only those with 3 or more citations were described in detail in this review. On the other hand, due to limited scientific investigations about the antiasthmatic activity of the natural products from animal and microorganism sources, all studies that fit the inclusion criteria were described in the next sections.

### 2.1. Natural Products from Plants

The use of natural products obtained from plants by the traditional medicine has been reported from centuries, especially in countries as China, Japan, and India [212]. Thus, the topics below concern these products or bioactive compounds originated from the most studied plants used on asthma therapy.

#### 2.1.1. Flavonoids

Flavonoids are natural compounds from plants, nuts, and fruits that are chemically characterized by the presence of two benzene rings (A and B) linked through a heterocyclic pyrene ring (C). They represent a large group of polyphenolic secondary metabolites [213] with more than 8,000 different compounds already identified [214]. Considering their chemical structure, they can be classified as flavans, flavanones, isoflavanones, flavones, isoflavones, anthocyanidins, and flavonolignans [214]. Flavans or isoflavans possess a heterocyclic hydrocarbon skeleton, chromane, and a substitution on its C ring, in carbons 2 or 3, by a phenyl group (B ring). Flavanones and isoflavanones show an oxo-group in position 4. The presence of a double bond between C2 and C3 indicates flavones and isoflavones, and the addition of a C1 to C2 double bond represents anthocyanidins [214].

The diversity in their chemical structure contributes to their broad range of physiological and biological activities, from which it can be highlighted the antioxidant, anti-inflammatory, antiallergic, antiviral, hepatoprotective, antithrombotic, and anticarcinogenic activities [213]. In this review, 14 studies reported flavonoids as a group of compounds able to be used on asthma treatment. The following subsections show the main flavonoids with antiasthmatic activity reported in the literature and used by the traditional medicine. These studies attributed the antiasthmatic activity of plant extracts containing these compounds, in part, due to their presence in the phytocomplex.

*(1) Flavone Compounds: Chrysin, Baicalin, Luteolin, and Oroxylin A*. Defined as 5,7-dihydroxy-2-phenyl-1-4*H*-chromen-4-one, chrysin is classified as a flavone that can be found in *Passiflora caerulea* and *Passiflora incarnate* flowers, as well as in *Matricaria chamomilla*, popularly known as chamomile, besides being present in propolis and other plants [90, 100]. Chrysin is a compound able to suppress the proliferation of airway smooth muscle cells as well as to promote a reduction in the IL-4, IL-13, IgE, and interferon-γ levels that lead to an attenuation in the asthma inflammatory process [89]. Bae et al. [90] performed their studies through an *in vitro* cell culture model with the purpose to describe how the chrysin was able to promote the inhibitory effect in the proinflammatory cytokines. They suggested that this effect was caused by the intracellular calcium reduction in mast cells, since calcium is responsible for proinflammatory cytokine gene transcription [90]. In addition, a study performed by Yao and colleagues [88] investigated the activity of chrysin against asthma in mice sensitized with ovalbumin (OVA). Their results revealed that chrysin would be a promising compound able to be used for controlling airway remodeling and clinical manifestations of asthma [88].

Baicalin, a 7-glucuronic acid-5,6-dihydroxyflavone, is a natural metabolite easily found in leaves and barks from several species of the *Scutellaria* genus [215]. Studies performed by Park and colleagues [208] investigated the anti-inflammatory activity of baicalin using an asthma-induced animal model. The results showed that this compound decreased the inflammatory cell infiltration and the levels of TNF-*α* in the bronchoalveolar lavage fluids (BALF). The activity of the baicalin was attributed to the fact that this metabolite selectively inhibits the enzyme activity of PDE4 and suppresses the TNF-*α* expression induced by the lipopolysaccharides on macrophages, indicating a potential use of this metabolite in asthma treatment [74].

Additionally, luteolin (2-(3,4-dihydroxyphenyl)-5,7-dihydroxy-4-chromenone), another compound that had also demonstrated antiasthma activity, is widely found in aromatic flowering plants, such as *Salvia tomentosa* and Lamiaceae, as well as in broccoli, green pepper, parsley, and thyme [216]. Shen and colleagues [133] studied its pharmacological activity through inhibition of the GABAergic system, which is responsible for the overproduction of mucus during the asthmatic crisis by overstimulation of the epithelial cells. The study indicated that this compound was able to promote the attenuation of the goblet cell hyperplasia by the partial inhibition of GABA activities [133].

Another antiasthmatic flavonoid compound is oroxylin A, a flavone found in the extract of *Scutellaria baicalensis Georgi* and *Oroxylum indicum* tree [156]. According to Zhou [157], oroxylin A, or 5-7-dihydroxy-6-methoxy-2-phenylchromen-4-one, was able not only to reduce the airway hyperactivity in an OVA-induced asthma murine model, but also to decrease the levels of IL-4, IL-5, IL-13, and OVA-specific IgE in BALF [157]. This study also showed the ability of oroxylin A in inhibiting the alveolar wall thickening in addition to avoid the inflammatory cell infiltration in the perivascular and peribronchial areas assessed by histopathological evaluation [157].

*(2) Flavonol Compounds: Quercetin, Galangin, and Kaempferol*. Quercetin (2-(3,4-dihydroxyphenyl)-3,5,7-trihydroxy-4H-chromen-4-one), a flavonol compound widely found in onions, apples, broccoli, cereals, grapes, tea, and wine, has been known as the main active compound of these plants and, therefore, responsible for their widespread use in traditional medicine for the treatment of inflammatory, allergic, and viral diseases [213]. The studies using this compound as antiasthma were performed in cell cultures and rats, as *in vitro* and *in vivo* models, respectively, showing its high capacity to reduce inflammatory processes. According to these studies, the anti-inflammatory mechanism of quercetin is attributed to the lipoxygenase and PDE4 inhibition and reduction on histamine and leukotriene release, which promote a decrease in the proinflammatory cytokine formation and production of IL-4, respectively. In addition, quercetin also promoted the inhibition of human mast cell activation by Ca^2+^ influx and prostaglandin release inhibition [182], favoring the therapeutic relief of the asthma symptoms and decreasing the short-acting *β*-agonist dependence [181, 182].

Galangin, a compound chemically defined as 3,5,7-trihydroxy-2-phenylchromen-4-one, easily found on *Alpinia officinarum* [217], had its pharmacological activity evaluated using a specific-pathogen-free mice model [115]. The study, performed by Liu [115], showed an effective response against the *in vivo* OVA-induced inflammation as well as a reduction on the ROS levels *in vitro.* Furthermore, galangin acted as an antiremodeling agent in asthma, since this compound inhibited the goblet cell hyperplasia, lowering the TGF-*β*1 levels and suppressing the expression of vascular endothelial grown factor (VEGF) and matrix metalloproteinase-9 (MMP-9) in BALF or lung tissue. This result highlighted its antiremodeling activity in the TGF-*β*1-ROS-MAPK pathway, proving its potential use on asthma treatment [115].

Another flavonol, kaempferol, chemically defined as 3,5,7-trihydroxy-2-(4-hydroxyphenyl)-4H-chromen-4-one, is widely found in citrus fruits, broccoli, apples, and other plant sources [213]. This compound has been studied due to its pharmacological potential, especially against inflammation. In the study performed by Chung et al. [127], an OVA-induced airway inflammation mouse model of asthma was performed, demonstrating that kaempferol can significantly reduce the inflammatory process due to the decrease of the inflammatory cell infiltration and the decrease of production of inflammatory cytokines and IgE antibodies. In addition, this compound was also able to reduce the intracellular ROS production in the airway inflammation reaction [127].

Furthermore, Mahat et al. [218] demonstrated that the anti-inflammatory activity of kaempferol occurs through the inhibition of nitric oxide and nitric oxide-induced COX-2 enzyme activation, further inhibiting the cytotoxic effects of nitric oxide, reducing the prostaglandin-E2 production [218]. To improve the possibility of the use of kaempferol as a bioactive on the development of new drugs or medicines, the previously mentioned study by Chung [127] also describes the antiasthma activity of a glycosylated derivative of kaempferol, the kaempferol-3-O-rhamnoside. The glycosylation of kaempferol improved its solubility and stability, besides reducing its toxicity [127], allowing the production of a compound with great potential to increase the asthma therapeutic arsenal. According to this rationale, this compound may be responsible for the anti-inflammatory properties of the plant extracts containing this substance and that have been used to asthma treatment.

#### 2.1.2. Resveratrol

Resveratrol is a natural stilbenoid compound, a class of polyphenol obtained from the bark of red fruits, with known antioxidant and promising anti-inflammatory and antiasthma activities [186]. In studies using eosinophils obtained from asthmatic individuals, Hu et al. [185] demonstrated that resveratrol induces not only cell cycle arrest in the G1/S phase, but also apoptosis, allowing a decrease in the eosinophil number [185], thus reducing the neutrophil migration and, consequently, preventing the histamine and PGD-2 release, avoiding vasodilatation, mucus production, and bronchoconstriction (Figure 1). Additionally, Lee and colleagues [129] demonstrated that resveratrol was effective against the asthmatic mouse model once this polyphenol induced a significant decrease in the plasma level of T-helper-2-type cytokines, such as IL-4 and IL-5. It also decreased the airway hyperresponsiveness, eosinophilia, and mucus hypersecretion [184]. Although performed by different methods, the studies are in agreement regarding the scientific evidence that supports the use of resveratrol by oral route as an effective natural compound to treat asthma patients.

#### 2.1.3. *Boswellia*

*Boswellia* is a tree genus that produces oil known as frankincense, which is obtained through incisions in the trunks of these trees. This oil is composed by 30–60% resin, 5–10% essential oils, and polysaccharides [219]. Studies performed using this product evaluated its pharmacological activities revealing that the *Boswellia* bioactives are boswellic acids and AKBA (3-O-acetyl-11-keto-*β*-boswellic acid), both responsible for preventing NF-*κ*B activation and, consequently, inhibiting IL-1, IL-2, IL-4, IL-6, and IFN-gamma release [52]. They also inhibit LOX-5, thus preventing leukotriene release [78]. Thus, based on the physiopathology of asthma, it is possible to infer that these compounds may act as antiasthma molecules from the tree genus, once these enzymes and mediators are involved in the asthma-related inflammation. Moreover, another study that aimed at evaluating the antiasthma activity of these compounds showed that the association between *Boswellia serrata*, *Curcuma longa*, and *Glycyrrhiza* had a pronounced effect on the management of bronchial asthma [79], suggesting its potential on asthma therapy.

### 2.2. Natural Products from Animal Source

Animal-derived natural products still represent the minority of natural sources for products intended for asthma treatment. Nonetheless, many studies describe the use of animal-based products, such as oils, milk, and spleen as a complementary therapy for several diseases, including asthma. The traditional medicine reports the benefits of consuming some animal parts and animal products, once they can be rich in compounds such as lipids, prostaglandins, unsaturated fatty acids, enzymes, and polysaccharides, which are responsible for their pharmacological activities [220, 221]. In addition, animal sources are also widely cited as biocompatible and biodegradable sources, suggesting their safe use. The animal products and compounds cited in this session can be obtained from several sources, such as mammals, amphibians, and crustaceans, demonstrating its wide range of possibilities.

#### 2.2.1. Animal Sea Source: *Holothuroidea, Penaeus*, and *Sarcophyton ehrenbergi*

Marine ecosystems represent an important source of natural compounds due to their wide biodiversity, which include animals and plants that are unique to this environment. Therefore, many studies have been performed to evaluate the antimicrobial, anti-inflammatory, antiviral, and antiasthmatic potential of algae and sea animals.

On this concern, the sea cucumber, a marine invertebrate animal that belongs to the class *Holothuroidea*, usually found in the benthic areas and deep seas, has been used by Asian and Middle Eastern communities in the traditional medicine as elixir, due to its pharmacological activity on the treatment of hypertension, asthma, rheumatism, cuts, burns, and constipation [188]. These pharmacological activities are attributed to the presence of saponins, cerebrosides, polysaccharides, and peptides on its composition [188, 220]. Bordbar et al. [220], in a literature review, mentioned an experimental study from Herencia et al. [222] in which sea cucumber extract showed a reduction of the enzymatic activity of cyclooxygenase in inflamed mice tissues, without promoting any modification on the cyclooxygenase enzyme, showing that the sea cucumber extract is a potent natural product able to be used against several inflammatory diseases [220].

Ozdemir and colleagues [87] investigated the pharmacological activity of chitin, a polysaccharide formed by repeated units of N-acetylglucosamine to form long chain through *β*-(1-4) linkage [221], the major compound of the shrimp (*Penaeus*) exoskeleton. In this study, the authors performed the intranasal administration of chitin microparticles in the asthma-induced mice model, which promoted the reduction of serum IgE and peripheral blood eosinophilia, besides the decrease of the airway hypersensitivity [87]. Additionally, another study identified and isolated ten new prostaglandin derivatives from the *Sarcophyton ehrenbergi* extract, a soft coral species found in the Red sea [194], from which five of them showed inhibitory activity against PDE4 (44.3%) at 10 *μ*g.mL^–1^, suggesting its utilization on asthma and chronic obstructive pulmonary disease treatment, once PDE4 is the drug target on the treatment of both diseases [194].

Finally, these studies demonstrated that marine source needs to be further investigated, since a wide variety of bioproducts and/or bioactives with potential anti-inflammatory activity and antiasthmatic proprieties can be found in this environment.

#### 2.2.2. Bullfrog *(Rana catesbeiana Shaw)* Oil

The bullfrog oil is a natural oil extracted from the adipose tissue of the amphibian *Rana catesbeiana* Shaw, which is originated from North America and has its meat widely commercialized around the world [223]. This oil has been used by the traditional medicine to treat inflammatory disorders, especially asthma [223]. This oil is composed of a mixture of mono- and polyunsaturated fatty acids and bile-derived steroid compound (ethyl iso-allocholate) [81, 224], which are responsible for its therapeutic properties [81].

According to Yaqoob [57], the presence of oleic, linolenic, stearic, palmitic, and myristic fatty acids can promote the suppression of immune cell functions [58]. Based on such evidence, it is possible to infer that the bullfrog oil, due to its chemical composition, can be used on the treatment of inflammation-related disorders such as asthma. However, further studies are needed to confirm this hypothesis.

#### 2.2.3. Other Products Derived from Animals

Although the majority of the currently used animal products by the traditional medicine for asthma treatment belong from animal tissues, there is evidence that mammal fluids, for example, buffalo spleen liquid, milk, and colostrum, can act on the immune system promoting the decrease of asthma symptoms [80].

The buffalo spleen liquid was investigated in a study performed by Neamati and colleagues [80], in which pigs were asthma sensitized using ovalbumin, followed by administration of the buffalo spleen liquid-based adjuvant. A decrease in the tracheal response as well as a reduction in the white blood cell number in lung lavage was observed on sensitized animals when compared to healthy animals [80], showing the potentiality of this fluid in promoting asthma control. In addition, another study was performed to evaluate the antiasthma activity using milk and colostrums, which contain linolenic acid and proteins like lactoferrin [141], as a natural product. This study showed a modulation in the plasma lipid concentration in human and animal models and a decrease in the allergic airway inflammation induced by ragweed pollen grain extract.

### 2.3. Bioactives Obtained from Microorganisms

The use of bacteria and fungi metabolites on the treatment of several diseases is widely reported since the penicillin discovery. However, more recent studies have further investigated the antiasthmatic potential of these metabolites [225]. On this concern, a study performed by Lu and colleagues [156] evaluated the antiasthma activity of the bacterial lysate OM-85 Broncho-Vaxom (BV), a patented pharmaceutical product [134]. The study observed that the bacterial lysate coupled with the conventional treatment was able to increase the rate of natural killer T cells on the peripheral blood, decreasing the cytokine level (cytokines type not described) and, then, promoting the reduction of asthma symptoms. Furthermore, kefir, a fermented milk drink produced by lactic and acetic acids from bacteria, which also presents the kefiran, an insoluble polysaccharide as main component [128, 129], had its *in vivo* anti-inflammatory activity evaluated. This compound was able to reduce at normal levels the release of IL-4, IL-6, and IL-10 along with the production of INF-*γ* and TNF-*α* [128]. In addition, the intragastric administration of kefiran promoted the reduction of OVA-induced cytokine production in a murine asthma model, decreasing the pulmonary eosinophilia and mucus hypersecretion [128, 129].

Therefore, based on these reports and historical facts regarding the use of microorganisms as source for isolation of new bioactives and the development of medicines, it is important to highlight that these new agents may contribute to the current asthma treatment.

## 3. Conclusion: Widely Used Active Pharmaceutical Ingredients from Natural Source

As previously demonstrated, natural products have been extensively used as a complementary treatment for asthma therapy. Studies concerning these products have aimed at investigating their activity as a matrix of compounds to complement or replace current asthma treatment, while others aim at isolating compounds to generate new medicines based on synthetic drugs of natural origin [226].

Historically, natural products have contributed tremendously to the development of marketable medicines to the treatment of several diseases [226]. The evaluation of their therapeutic activities and identification and isolation of their bioactive molecules allowed not only their clinical use, but also the discovery of the pharmacophore groups and the radicals responsible for their toxicity or their biopharmaceutics aspects. In fact, based on such studies, it is possible to perform structural or delivery changes on these compounds that would increase their safety or would be able to module their half-life allowing to target them to specific action sites [227].

This review shows the experimental studies that identified the antiasthma activity of different natural sources in the last decade, along with the molecules responsible for that. Altogether, these studies presented preliminary data that require further investigations about these compounds in order to, in a near future, be used on the production of designing medicines. Currently, a few natural-based active compounds are already available in the market, such as ipratropium bromide, theophylline, epinephrine, and sodium cromoglycate [226, 228–231].

Ipratropium bromide, an anticholinergic drug able to promote bronchodilation, has been widely used for the treatment of asthma. This compound was synthesized from atropine, a compound extracted for the first time in 1809 from *Atropa belladonna* L. However, it can be also found in other plants from the *Solanaceae* family [228, 229]. In spite of that, only in 1833, its chemical structure was elucidated, and in 1850, it was implemented for clinical use, allowing the proper understanding of its *in vivo* biopharmaceutics and therapeutic characteristics [232].

Theophylline is an antiasthmatic drug widely used in the management of severe persistent asthma, promoting the bronchodilation and attenuation of asthma inflammation. Also known as 1,3-dimethylxanthine, this molecule was extracted in 1888 from *Theobroma cacao* L. and *Camellia sinensis* L., plants presented in several countries. Later in 1922, this drug was introduced on asthma therapy [233]. Years after, epinephrine, also known as adrenaline, was extracted from the *Ephedra sinica*, a plant widely used in the Chinese traditional medicine, allowing the synthesis of beta-agonist antiasthmatic drugs, such as salbutamol and salmeterol, currently used in asthma treatment [226].

Furthermore, sodium cromoglycate, a drug obtained from the khellin bioactive extracted from *Ammi visnaga* (L) *Lamk*, has been used as a bronchodilator based on its ability of inhibiting mast cell degranulation, which enabled its use on the asthma treatment [226, 230].

Overall, these reports highlight the relevance of the investigation and isolation of new bioactive compounds that could present antiasthmatic potential. As the current asthma treatment involves drugs that have been extensively studied in the past decades, the experimental studies that evaluate the activity of compounds obtained from diverse natural sources might allow the development of new antiasthmatic drugs in the near future.

## 4. Final Considerations

The current asthma treatment is of high cost and has many side effects, which compromises the patient treatment compliance. Literature reports show that asthma treatment can be improved using natural products to complement the traditional drugs, since those products are of low cost and biocompatible and show reduced side effects. The literature search included the keywords asthma, natural products, and treatment, individually, resulted in 14,296,762 studies, including scientific articles, reviews, editorial reference works, and abstracts. Additionally, the keyword combination “Asthma + Natural Products” found 18,111 studies, “Asthma + Treatment,” 209,423 studies, “Natural Products + Treatment,” 459,685 studies, and “Asthma + Treatment + Natural Products,” 1,986 studies. Thus, after screening for duplicate studies, 1,934 abstracts were evaluated. Finally, based on the inclusion criteria, 172 studies reporting the use of natural products on asthma treatment were included in this review, summarizing a total of 160 studies that reported plants as natural source, 9 from animal source, and 3 studies describing bacteria and fungi as bioactive sources, totalizing 134 compounds which can be used as complementary or alternative medicine on asthma treatment. Plants were found to be the major source of products used by the folk medicine to treat asthma, since they are a renewable source of easy access. Also, due to their variety of secondary metabolites, plants are able to promote antiasthma activity mainly due to their anti-inflammatory and bronchodilator properties. This study revealed that flavonoids, phenolic acids, and terpenoids are the main elucidated compounds able to promote the attenuation of asthma symptoms. On the other hand, a lack of scientific reports regarding the pharmaceutical activity of natural products from animal and microorganism sources has limited their use. However, these products still represent an important source of bioactive compounds able to be used on asthma treatments. In addition, despite the relevant antiasthmatic activity, the literature search showed a lack of investigations concerning the pharmacokinetics properties as well as more accurate information regarding efficacy, safety, and the required dosage to induce *in vivo* antiasthma activity. In conclusion, due to the fact that current asthma treatment involves drugs obtained from natural products widely explored in the past, the current experimental studies reported in this review may lead to the development of new drugs in the future, able to improve the antiasthmatic treatment.

## Figures and Tables

**Table 1:** List of natural compounds described in the literature reviewed.

Product	Product form	Product source	Active compound	Compound class/type	Mechanism of action	Reference
1,8-Cineol	Isolated compound	Essential oil of *Eucalyptus globulus* leaves	1,8-Cineol	Monoterpene	Reduces the expression of NF-*κ*B target gene MUC2	Greiner et al. [62]

3-Methoxy-catalposide	Isolated compound	*P. rotundum* var*. subintegrum* extract	3-Methoxy-catalposide	Iridoid glycoside	Inhibits the expression of cyclooxygenase (COX)-2, nitric oxide synthase (iNOS), and proinflammatory genes (IL-6, IL-1*β*, and TNF-*α*)	Ryu et al. [63]

*Achyranthes aspera* L	Ethanolic extract	Roots	Not reported	Not reported	Bronchoprotective activity	Dey [64]

*Ailanthus excelsa Roxb*	Aqueous extract	Barks	Not reported	Not reported	Bronchodilator and mast cell stabilizing activities	Kumar [65]

*Allium Cepa* L. and quercetin	Extract and isolated compound	Methanolic extract and vegetable	Quercetin [2-(3, 4-dihydroxyphenyl)-3, 5, 7-trihydroxy-4H-1-benzopyran-4-one, 3, 3′, 4′, 5, 6-entahydroxyflavone]	Flavonoid	Reduce the production of proinflammatory cytokines (IL-4, IL-5, IL-13) and promote the relaxation of tracheal rings	Oliveira et al. [66]

*Alstonia scholaris* (L.) R. Br.	Extract	Leaves of *Alstonia scholaris (*L.*) R. Br*.	Scholaricine, 19-epi-scholaricine, vallesamine, picrinine	Alkaloid	Reduce the eosinophilia, the production of proinflammatory cytokine (IL-4) and the expression of serum IgE and eotaxin	Zhao et al. [67]

*Amorphophallus konjac (konjac)*	Gel extract	Not reported	Not reported	Plant	Not elucidated	Chua et al. [68]

*Andropogon muricatus*	Crude extract	Aerial parts	Vetivenes. vetivenol, vetivenic acid, and vetivenyl acetate	Sesquiterpenic compounds	Inhibit the Ca^2+^ channels and phosphodiesterase activity	Shah and Gilani [69]

*Anoectochilus formosanus Hayata*	Aqueous extract	Whole plant	Kinsenoside	Plant	Reduce the IL-4 production by Tregs and enhance the production of IL-12 and IFN-*γ* by Th1 differentiation	Hsieh et al. [70]

*Artemisia maritima*	Essential oil	Leaves	1,8-Cineol, camphor, camphene, and *β*-caryophyllene	Terpenoid	Inhibit the Ca^2+^ channels and phosphodiesterase activity	Shah et al. [71]

*Aster tataricus* L. f.	Extract	Rhizomes	Kaempferol, aurantiamide, and astin C	Flavonoid	Inhibit the expression of NF-*κ*B and promote the activation of beta-2 adrenergic receptor	Chen and Zheng [72]
*Aster yomena* (Kitam.) Honda	Ethanolic extract	Leaves	Phenolic compounds not specified	Phenolic compounds	Attenuate the production of NO and IL-1*β*, and suppress the expression of NF-*κ*B. In addition, suppress the activation of TLR4 and promote a reduction of intracellular ROS production	Kang et al. [73]

Baicalin	Isolated compound	Leaves and branch	7-Glucuronic acid-5,6-dihydroxyflavone	Flavonoid	Suppress the lipopolysaccharide-induced TNF-*α* expression and inhibit the cyclic adenosine monophosphate-specific phosphodiesterase 4 (PDE4)	Park et al. [74]

*Baliospermum montanum Müll. Arg. (Euphorbiaceae)*	Chloroformic and ethanolic extracts	Leaves	Alkaloids, triterpenoids, diterpenoids, and glycosides	Alkaloids, triterpenoids, diterpenoids, and glycosides	Stabilize the mast cell degranulation and decrease the histamine release	Venkatesh et al. [75]

Berry fruit	Polyphenolic extract	Not reported	Phenolic compounds not specified	Phenolic compounds not specified	Not reported	Power et al. [76]

*Boswellia serrata, Boswellia carterii*, *and frankincense*	Essential oil	Resinous part	Fl-boswellic acid, acetyl-fl-boswellic acid, 11-keto-fl-boswellic acid, and acetyl-11-keto-fl-boswellic acid	Boswellic acids	Inhibition of leukotriene biosynthesis	Hamidpour et al. [77] and Al-Yasiry and Kiczorowska [78]

*Boswellia serrata, Glycyrrhiza glabra*, *and Curcuma longa*	Essential oil extract and extract	Resinous part, licorice root and turmeric root, respectively	Curcumin and fl-boswellic acid	Polyphenol	Reduce the plasma level of the leukotriene C4, nitric oxide and malondialdehyde	Houssen et al. [79]

Buffalo spleen lipid and a bacterial polypeptide	Extract	Animal-derived and microorganism-derived, respectively	Not reported	Not reported	Reduce the tracheal responsiveness and the amount of white blood cells	Neamati et al. [80]

Bullfrog oil *(Rana catesbiana* shaw)	Oil	Bullfrog adipose tissue	Oleic, linolenic, stearic, palmitic, and myristic acids. Eicosapentaenoic acids and decosahexaenoic acid	Fatty acids	Not elucidated	Amaral-Machado et al. [81]

*Bu-zhong-yi-qi-tang*	Aqueous extract	Root of Astragalus mongholicus Bunge, Panax ginseng C.A.Mey, Angelica dah-rica Fisch. Ex Hoffm and Bupleurum chinense DC. Rhizome of Z*ingiber officinale* Rosc, Atractylodes macrocephala Koidz, and *Cimicifuga foetida L*. Fruit of Ziziphus jujuba Mill. var. inermis Rehd. Pericarp of Citrus reticulata Blanco. Root and rhizome of *Glycyrrhiza uralensis* Fisch	Not reported	Not reported	Reduce the level of eotaxin, Th2-related cytokines (IL-4, IL-5, IL-13), IgE, and eosinophilia	Yang et al. [82]

*Caenorhabditis elegans*	Crude extract	Microorganism	Not reported	Not reported	Modulate the immunologic Th1/Th2 response	Huang et al. [83]

*Camellia sinensis* L.	Aqueous extract	Not reported	Polyphenois and flavonoids	Polyphenois and flavonoids	Not elucidated	Sharangi [84]

*Carica papaya*	Extract	Leaves	Tanins, alkaloids, steroids, and quinones	Tanins, alkaloids, steroids, and quinones	Reduce the expression of IL-4, IL-5, eotaxin, TNF-*α*, NF-*κ*B, and iNOS	Elgadir et al. [85]

*Carum roxburghianum*	Crude extract	Seeds	Hydrocarbons, wax esters, sterol esters, triacylglycerols, free fatty acids, diacylglycerols, lysophosphatidylethanolamines, and phosphatidylinositols	Hydrocarbons, wax esters, sterol esters, triacylglycerols, free fatty acids, diacylglycerols, lysophosphatidylethanolamines, and phosphatidylinositols	Bronchodilator activity	Khan et al. [86]

Chitin	Isolated compound	Shrimp	Chitin	Polysaccharide	Not elucidated	Ozdemir et al. [87]

Chrysin	Isolated compound	Marketable synthetic compound	5,7-Dihydroxy-2-phenyl-1-4H-chromen-4-one	Flavonoid	Reduces the histamine release and decreases the gene expression of proinflammatory cytokines (IL-1*β*, IL-4, IL-6, TNF-*α*, NF-*κ*B)	Yao et al. [88]; Yao et al. [89]; Bae et al. [90]
*Cissampelos sympodialis Eichl*	Extract	Leaves	Warifteine	Alkaloid	Reduce the expression of IL-3 and IL-5, increase the IL-10 level, and decrease the density of inflammatory cells	Cerqueira-lima et al. [91]

*Citrus tachibana*	Ethanolic extract	Leaves	Coumarins, carotenoids, and flavonoids	Coumarins, carotenoids, and flavonoids	Modulate the Th1/Th2 imbalance by inhibition of NF-*κ*B signaling and histamine secretion	Bui et al. [92]

Conjugated linoleic acid	Conjugated compound	Fatty tissue from ruminants	Cis, cis-9,12-octadecadienoic acid	Polyunsaturated fatty acid	Modulate the PPARγ-dependent and PPARγ-independent inflammation signaling, the eicosanoid production, and humoral immune response	Macredmond and Dorscheid [93]

Coumarins	Isolated compound	Synthetic compounds	6,7-Dihydroxycoumarin, 7-hydroxycoumarin and 4-methyl-7-hydroxycoumarin	Coumarin	Not elucidated	Sanchez-Recillas et al. [94]

Crocetin	Isolated compound	Marketable synthetic compound	Crocetin	Carotenoid	Activates the FOXP3 signaling through TIPE2	Ding et al. [95]

Curcumin	Isolated compound	Curcuma longa	(1E, 6E)-1,7-Bis (4-hydroxy- 3-methoxyphenyl)-1,6- heptadiene-3,5-dione	Polyphenol	Inhibits the Notch1-GATA3 signaling pathway	Zheng et al. [96]; Chong et al. [97]

Cyclotheonamides	Isolated compound	Marine	Not reported	Cyclic pentapeptides	Inhibit the human *ß*-tryptase	Schaschke and Sommerhoff [98]

Diallyl-disulfide	Isolated compound	Garlic oil	Diallyl-disulfide	Organosulfur	Activates the NrF-2/HO-1 pathway and suppresses the NF-*κ*B	Shin et al. [99]

Dietary plant stanol esters	Not reported	Fatty acid	Not reported	Stanol ester	Reduce the total plasma IgE, IL-1*β*, IL-13, and TNF-*α*	Brull et al. [100]

*Dioscorea nipponica*	Isolated compound	Not reported	Diosgenin	Steroidal saponin	Suppress the secretion of TNF-*α*, IL-1*β*, and IL-6	Junchao et al. [101]

D-*α*-tocopheryl acetate	Isolated compound	Natural source	D-*α*-tocopheryl acetate	Vitamin	Inhibits the oxidative stress. Modulates the allergic inflammation and the airway hyperresponsiveness	Hoskins et al. [102]

*Echinodorus scaber*	Hydroethanolic extract	Leaves	Vitexin, rutin, and gallic acid	Phenolic compounds	Decrease the migration of inflammatory cells and reduce the Th2 cytokines and IgE levels	Rosa et al. [103]

*Eclipta prostrata* (L.)*L*.	Methanolic extract	Whole plant	Wedelolactone and demethylwedelolactone	Coumestan	Reduce the bronchial hyperresponsiveness and the production of Th2 cytokines	De Freitas Morel et al. [104]

*Ecklonia cava*	Marine alga	Brown macroalgae	Fucodiphloroethol and phlorofucofuroeckol A	Phlorotannins	Downregulate the Fc*ε*RI expression and block the IgE-Fc*ε*RI binding	Vo et al. [105]

*Ephedra intermedia*	Crude extract	Aerial parts	Ephedrine and pseudoephedrine	Alkaloids	Not elucidated	Gul et al. [106]

Ellagic acid	Isolated compound	Marketable synthetic compound	Ellagic acid	Polyphenol	Inhibits the activation of the NF-*κ*B	Zhou et al. [107]

Emodin	Isolated compound	Roots and barks of *Rheum palmatum* and *Polygonum multiflorum*	1,3,8-Trihydroxy-6-methylanthraquinone	Anthraquinone	Suppresses the characteristics of airway inflammation, mucin components, and chitinase protein expression. Inhibits the NF-*κ*B signaling pathway	Shrimali et al. [108]

*Euphorbia hirta*	Aqueous extract	Not reported	Galloylquinic acid, phorbol acid, leucocyanidol, quercitol, camphol, quercetin, chlorophenolic acid, shikimic acid	Tanins, leucoanthocyanidins, flavonoids, and phenolic compounds	Not elucidated	Kunwar et al. [109]

Sesame	Fixed oil	Seeds	5,5′-(1S, 3aR, 4S, 6aR)-Tetrahydro-1H, 3H-furo [3,4-c]furan-1,4-diylbis-1,3-benzodioxole	Polyphenol	Decreases the levels of IL-4, Il-5, IL-13, and serum IgE. Reduces the amount of inflammatory cells and the eosinophil infiltration	Lin et al. [41]

Farnesol	Isolated compound	Fruits, leaves, flowers	3,7,11-Trimethyl-2,6,10-dodecatrien-1-ol	Sesquiterpene	Increases the level of IgG2a/IgE and reduces the total IgE, IgA, IgM, IgG	Ku and Lin [110]

Feverfew *(Tanacetum parthenium* L.)	Extract	Leaves and parts above the ground	Parthenolide	Sesquiterpene	Inhibit the I*κ*B kinase complex and the histamine release	Pareek et al. [111]

Flavonoids	Isolated compound	Vegetables (capers, tomatoes, fennel, sweet potato leaves, etc.), fruits (apple, apricots, grapes, plums, and berries), cereals (green/yellow beans and buckwheat)	Not reported	Polyphenol	Prevent the IgE synthesis and the mast cell degranulation. Reduce the airway hyperresponsiveness and inhibit the human phospholipase A2	Castell et al. [112]; Lattig et al. [113]

*Fumaria parviflora Linn*	Aqueous methanolic extract	Aerial parts	Fumarophycine, cryptopine, sanactine, stylopine, bicuculline, adlumine, perfumidine, and dihydrosanguirine	Alkaloids	Block the muscarinic receptors and the Ca^2+^ channels	Najeeb ur et al. [114]

Galangin	Synthetic compound	*Alpinia officinarum*	3,5,7-Trihydroxy-2-phenylchromen-4-one	Flavonol	Inhibits the TGF-*β*1 signaling by ROS generation and MAPK/Akt phosphorylation	Liu et al. [115]

*Geastrum saccatum*	Solid extract	Fruiting bodies of *Geastrum saccatum*	*β*-Glucose	Polysaccharide	Inhibit the NOS and COX	Guerra dore et al. [116]

Ginsenosides	Synthetic compound	Root of ginseng	Ginsenosides	Glycoside	Suppress the IL-4 level, increase the production of IFN-*γ*, and inhibit the mucus overproduction and recruitment of eosinophils	Chen et al. [117]

Grape seed	Extract	Seeds	Not reported	Not reported	Not elucidated.	Mahmoud [118]

*Gymnema sylvestre R. Br.*	Extract	Leaves	Not reported	Tanins and saponins	Not elucidated.	Tiwari et al. [119]; Di Fabio et al. [120]

*Herba epimedii*	Extract	Leaves	Icariin	Flavonoids, iridoid glycosides, and alkaloids	Inhibit the mRNA expression of TGF-*β*1 and TGF-*β*2. Modulate the TGF-*β* signaling	Tang et al. [121]

Higenamine	Isolated compound	*Tinospora crispa*, *Nandina domestica* T_HUNBERG_, *Gnetum parvifolium* C.Y. Cheng, *Asarum heterotropoides*	1-[(4-Hydroxyphenyl)methyl]-1,2,3,4-tetrahydroisoquinoline-6,7-diol	Alkaloid	Not elucidated	Zhang et al. [122]

Homoegonol	Isolated compound	*Styrax japonica*	3-[2-(3,4-Dimethoxyphenyl)-7-methoxy-1-benzofuran-5-yl]propan-1-ol	Lignan	Reduces the inflammatory cells count and Th2 cytokines	Shin et al. [123]

*Hypericum sampsonii*	Isolated compound	Aerial parts	Not reported	Polycyclic polyprenylated acylphloroglucinols	Not elucidated	Zhang et al. [124]

*Justicia pectoralis*	Extract	Aerial parts	7-Hydroxycoumarin	Coumarin	Decrease the tracheal hyperresponsiveness and the IL-1*β* and TNF-*α* levels	Moura et al. [125]

*Juniperus excelsa*	Crude extract	Aerial parts	(+)-Cedrol, (+)-Sabinene, (+)-limonene, terpinolene, endo-fenchol, cis-pinene hydrate, *α*-campholena, camphor, borneol, triene cycloheptane 1,3,5-trimethylene, *β*-myrcene, o-allyl toluene	Anthraquinones, flavonoids, saponins, sterol, terpenoids, and tanins	Inhibit the Ca^2+^ influx and the phosphodiesterase activity	Khan et al. [126]

Kaempferol	Isolated compound	Biotransformation of synthetic kaempferol by genetically engineered *E. coli*	Kaempferol-3-O-rhamnoside	Flavonoid	Reduces the inflammatory cells number, suppresses the production of Th2 cytokines and TNF-*α*	Chung et al. [127]

Kefir	Isolated compound	Kefir grains	Kefiran	Microorganism derived	Reduces the inflammatory cell number and decreases the level of IL-4, IL-13, IL-5, and IgE	Kwon et al. [128]; Lee et al. [129]

*Laurus nobilis* L.	Isolated compound	Leaves of *Laurus nobilis* L	Magnolialide	Sesquiterpene	Inhibit the mast cell degranulation and reduce the IL-4 and IL-5 production	Lee et al. [130]

*Lepidium sativum*	Crude extract	Seeds	Ascorbic acid, linoleic acid, oleic acid, palmitic acid, stearic acid	Vitamin and fatty acids	Promote a anticholinergic effect, inhibit the Ca^2+^ influx, and inhibit the phosphodiesterase activity	Rehman et al. [131]

L-Theanine	Isolated compound	Green tea of *Camellia sinensis*	L-Theanine (N-ethyl-L-glutamine)	Amino acid	Reduces the ROS production and decreases the levels of NF-*κ*B and MMP-9	Hwang et al. [132]

Luteolin	Isolated compound	*Perilla frutescens*	(2-(3,4-Dihydroxyphenyl)-5,7-dihydroxy-4-chromenone)	Flavonoid	Inhibits the mucus overproduction and the GABAergic system	Shen et al. [133]

Lysate bacterial (OM-85 Broncho-Vaxom)	Extract	*H. influenzae*, *S. pneumoniae*, *Klebsiella pneumoniae*, smelly nose *Klebsiella*, *S. aureus, Streptococcus pyogenes, Streptococcus viridans, Neisseria catarrhalis*	Not reported	Not reported	Increase the level of IL-4, IL-10, and IFN-*γ*	Lu et al. [134]

*Mangifera indica* L. *extract* (Vimang®)	Extract	Stem bark	Mangiferin (1,3,6,7-tetrahydroxyxanthone-c2-b-D-glucoside)	Xanthone	Inhibit the IgE production, the histamine release, and mast cell degranulation. Decrease the MMP-9 activity	Rivera et al. [135]
	Aqueous extract	Barks	Mangiferin (1,3,6,7-tetrahydroxyxanthone-c2-b-D-glucoside)	Xanthone	Reduce the inflammatory cells recruitment and the airway hyperresponsiveness. Increase the Th2 cytokines and attenuated the increase of the PIK3 activity	Alvarez et al. [136]

Mangosteen	Isolated compound	*Garcinia mangostana Linn.*	*α*- and *γ*-mangostin	Xanthone	Inhibits the histamine release and modulates the cytokine production	Jang et al. [137]

Marine bioactives	Isolated compound	Marine sponges *Petrosia contignata* and *Xestospongia bergquisita*	Contignasterol and xestobergsterol	Steroids	Upregulation of TNF-*β* and IL-10 expression	D'Orazio et al. [138]

Marshallagia marshalli	Isolated compound	Marshallagia marshalli	Secretory/excretory antigen	Microorganism derived	Prevent the release of TNF-*α* and IL-1*β*. Suppress the neutrophil migration	Jabbari et al. [139]

*Mikania laevigata* and *M. glomerata*	Extract	Leaves	Dihydrocoumarin, coumarin, spathulenol, hexadecanoic acid, 9, 12-octadecadienoic acid, 9,12,15-octadecatrineoic acid, cupressenic acid, kaurenol, kaurenoic acid, isopropyloxigrandifloric acid, isobutyloxy-grandifloric acid	Coumarins, terpenoids, steroids, and flavonoids	Not elucidated	Napimoga and Yatsuda [140]

Milk and colostrum	Conjugated compound	Bovine milk	Conjugated linoleic acid	Fatty acid	Modulate the cytokine and antibodies (IgE, IgM) production, interferon NO synthesis and iNOS activity. Modulate the mast cell degranulation	Kanwar et al. [141]

Monoterpenes	Isolated compound	Essential oil of several medicinal plants (*Matricaria recutita, Boswellia carterii, Pelargonium graveolens, Lavandula angustifolia, Citrus limon, Melaleuca alternifolia, Melaleuca viridiflora, Santalum spicatum, Cedrus atlantica*, and *Thymus vulgaris*)	Hydroxydihydrocarvone, fenchone, *α*-pinene, (S)-cis-verbenol, piperitenone oxide, *α*-terpinene, *α*-terpineol, terpinen-4-ol, *α*-carveol, menthone, pulegone, geraniol, citral, citronellol, perillyl alcohol, perillic acid, *β*-myrcene, carvone, limonene, thymol, carvacrol, linalool, linalyl acetate, borneol, l-borneol, bornyl acetate, terpineol, thymoquinone, thymohydroquinone, 1,8-cineol, l-menthol, menthone, and neomenthol	Terpenoids	Reduce the expression of NF-*κ*B target gene MUC2	Cassia et al. [142]

*Mandevilla longiflora*	Hydroethanolic extract	Plant xylopodium	Ellagic acid, hesperidin, luteolin, naringin, naringenin, and rutin	Polyphenol and flavonoids	Decrease the eosinophils, neutrophils, and mononuclear cell migration in BALF and by histopathological analysis. Decrease the IL-4, IL-5, IL-13, IgE, and LTB_4_ levels	Almeida et al. [143]

*Morus alba L*.	Isolated compound	Root bark	Moracin M. (5-(6-hydroxy-1-benzofuran-2-yl)benzene-1,3-diol)	Not reported	Inhibit the PDE4	Chen et al. [144]

*Haemanthus coccineus*	Extract	Dried bulbs	Narciclasine	Alkaloid	Inhibit the edema formation, the leucocyte infiltration, and cytokine synthesis in vivo. Block the interaction between the leucocyte and endothelial cells, the activation of isolated leucocytes (cytokine synthesis and proliferation) and of primary endothelial cells (adhesion molecule expression) in vitro. Suppress the NF-*κ*B-dependent gene transcription	Fuchs et al. [145]
Naringin	Isolated compound	Common grapefruit	Naringin	Flavone	Attenuates the bronchoconstriction by reduction of calcium influx	Wang et al. [146]

*Nielumbo nucifera*	Extract	Leaves	Nuiciferine and aporphine	Alkaloids	Attenuate the bronchoconstriction by reduction of calcium influx	Yang et al. [147]

Nigella sativa	Oil	Seeds	Thymoquinone (2-isopropyl-5-methyl-1,4-benzoquinone)	Quinone	Decrease the NO and IgE levels. Increase the IFN-*γ*	Salem et al. [148]; Koshak et al. [149]

NujiangexanthoneA	Isolated compound	Leaves of *Garcinia nujiangensis*	1,2,5,6-Tetrahydroxy-3-methoxy-4,7,8-tri(3-meth-ylbut-2-enyl)-xanthone	Xanthone	Suppresses the IgE/Ag activation and degranulation of mast cell. Suppresses the production of cytokines and eicosanoids, through inhibiting Src kinase activity and Syk-dependent pathways. Inhibits the release of histamine, PGD2 and leukotriene C4 generation. Inhibits the increase of IL-4, IL-5, IL-13, and IgE levels. Inhibits the cell infiltration and increases mucus production	Lu et al. [150]

Oleanolic acid	Synthetic compound	*Forsythia viridissima*	Oleanolic acid	Triterpenoid	Modulates the transcription factors T-bet, GATA-3, ROR*γ*t, and Foxp3	Kim et al. [151]

Omega 3	Isolated compound	Fish oil	*n* – 3 Polyunsaturated fatty acid	Fatty acid	Decreases the IL-17 and TNF-*α* levels	Hansen et al. [152]; Farjadian et al. [153]

Organic acids	Isolated compound	*Berberis integerrima* and *B*. *vulgaris* fruits	Malic, citric, tartaric, oxalic, and fumaric acids	Organic acids	Inhibits the Th2 cytokines	Ardestani et al. [154]; Shaik et al. [155]

Oroxylin A	Isolated compound	*Scutellariae radix*	5′7-Dihydroxy-6-methoxy-2phenyl-4H-1-benzopyran-4-one	Flavonoid	Reduces the airway hyperactivity. Decreases the levels of IL-4, IL-5, IL-13 and IgE in BALF	Lu et al. [156]; Zhou et al. [157]

Oxymatrine	Isolated compound	Root of the S*ophora* flavescens Aiton (Fabaceae)	Oxymatrine	Alkaloid	Inhibits the eosinophil migration, IL-4, IL-5, IgE, and IL-13 levels. Inhibits the expression of CD40 protein	Zhang et al. [158]

*P. integerrima Gall* and *Pistacia integerrima stew. Ex brand*	Methanolic and crude extract	Galls and whole plant	Not reported	Carotenoids, terpenoids, catechins, and flavonoids	Attenuate the TNF-*α*, IL-4, and IL-5 expression levels, and pulmonary edema by elevation of AQP1 and AQP5 expression levels	Rana et al. [159]; Bibi et al. [160]

*Paeonia emodi royle*	Extract	Rhizomes	1*β*, 3*β*, 5*α*, 23, 24-Pentahydroxy-30-nor-olean-12, 20(29)-dien-28-oic acid; 6*α*, 7*α*-epoxy-1*α*, 3*β*, 4*β*, 13*β*-tetrahydroxy-24, 30-dinor-olean-20-ene-28, 13*β*-olide; paeonin B; paeonin C; methyl grevillate; 4-hydroxy benzoic acid, and gallic acid	Terpenoids and phenolic compounds	Inhibits the lipoxygenase activity	Zargar et al. [161]

*Petasites japonicus*	Extract	Leaves	Petatewalide B	Not reported	Inhibit the degranulation of *β*-hexosaminidase in mast cells, the iNOS induction, and the NO production. Inhibits the accumulation of eosinophils, macrophages, and lymphocytes in BALF	Choi et al. [162]

*Peucedanum praeruptorum dunn*	Extract	Roots	Dihydropyranocoumarin, linear furocoumaris, and simple coumarin	Coumarins	Attenuate the airway hyperreactivity and Th2 responses	Xiong et al. [163]

Peucedani Radix	Extract	Roots	Nodakenin, nodakenetin, pteryxin, praeruptorin A, and praeruptorin B	Not reported	Inhibit the Th2 cell activation	Lee et al. [164]

*Eryngium*	Extract	Leaves, fruits, and roots	A1-barrigenol, R1-barrigenol, tiliroside, kaempferol 3-O-*β*-D-glucosyde-7-O-*α*-L-rhamnoside, rutin, agasyllin, grandivittin, aegelinol benzoate, aegelinol, R-(+)-rosmarinic (61), and R-(+)-3′-O-*β*-D-glucopyranosyl rosmarinic acid	Phenol, flavonoids, tannins, and saponins	Not elucidated	Erdem et al. [165]

*Pericampylus glaucus*	Extract	Stems, leaves, roots, and fruits	Periglaucine A-D and mangiferonic acid	Alkaloids, terpenoids, isoflavones, and sterols	Inhibit the COX enzymes activity	Shipton et al. [166]

*Aquilaria malaccensis*	Ethanolic extract	Seeds	Aquimavitalin	Phorbol ester	Inhibit the mast cell degranulation	Korinek et al. [167]

Phytochemicals	Isolated compound	Several medicinal plants	Luteolin, kaempferol, quercetin, eudesmin, magnolin, woorenoside, zerumbone, aucubin, triptolide, nitocine, berberine, and piperine	Flavonoids, lignans, terpenoids, and alkaloids	Suppress the TNF-*α* expression	Iqbal et al. [168]

*Picrasma quassioides* (D.Don) Benn	Alcoholic extract	Not reported	4-Methoxy-5- hydroxycanthin-6-one	Alkaloid	Decreases the inflammatory cell count in BALF. Reduces the IL-4, IL-5, IL-13, and IgE levels. Reduces the airway hyperresponsiveness. Attenuates the recruitment of inflammatory cells and the mucus production in the airways. Reduces the overexpression of inducible nitric oxide synthase (iNOS)	Shin et al. [169]

*Pinus maritime* (Pycnogenol®)	Extract	Barks	Procyanidin	Flavonoid	Decrease the NO production, the inflammatory cell count, and the levels of IL-4, IL-5, IL-13, and IgE in BALF or serum. Reduces the IL-1*β* and IL-6 levels, the expression of iNOS and MMP-9. Enhances the expression of heme oxygenase (HO)-1. Attenuates the airway inflammation and mucus hypersecretion	Shin et al. [170]

Ping chuan ke li					Not elucidated	Wang et al. [171]

Piperine	Isolated compound	*Piper nigrum* (black pepper) and *Piper longum* (long pepper)	Piperine	Alkaloid	Inhibits eosinophil infiltration and airway hyperresponsiveness by suppressing T cell activity and Th2 cytokine production	Chinta et al. [172]

Piperlongumine	Isolated compound	*Piper longum*	Piperlongumine (5,6-dihydro-1-[(2E)-1-oxo-3-(3,4,5-trimethoxyphenyl)-2-propenyl]-2(1H)-pyridinone)	Alkaloid	Inhibits the activity of inflammatory transcription factors, NF-*κ*B, and signal transducer and activator of transcription (STAT)-3 as well as the expression of IL-6, IL-8, IL-17, IL-23, matrix metallopeptidase (MMP)-9, and intercellular adhesion molecule (ICAM)-1. Suppresses the permeability and leukocyte migration, the production of TNF-*α*, IL-6, and extracellular regulated kinases (ERK) 1/2 along with the activation of NF-*κ*B	Prasad and Tyagi [173]

*Piper nigrum*	Ethanolic extract	Not reported	Piperine	Alkaloid	Inhibit the Th2/Th17 responses and mast cell activation	Bui et al. [174]; Khawas et al. [175]

*Plectranthus amboinicus (Lour.) spreng.*	Ethanol, methanol, and hexane extracts	Aerial parts	Rosmarinic acid, shimobashiric acid, salvianolic acid L, rutin, thymoquinone, and quercetin	Flavonoids	Not elucidated	Arumugam et al. [176]

Podocarpus sensu latissimo	Extract	Barks	3-Methoxyflavones and 3-O-glycosides	Flavonoids	Provinol and flavin-7	Abdillahi et al. [177]

Polyphenols and their compounds	Isolated compound	Provinol and flavin-7	Quercetin and resveratrol	Polyphenol	Decrease IL-4 and IL-5 levels, the airway hyperresponsiveness, and mucus overproduction	Joskova et al. [178]

Propolis	Isolated compound	Honey bees from several plants	Pinocembrin and caffeic acid phenethyl ester	Polyphenol and terpenoids	Inhibits TGF-*β*1	Kao et al. [179]

*Psoralea corylifolia*	Extract	Fruits	7-O-Methylcorylifol A, 7-O-isoprenylcorylifol A, and 7-O-isoprenylneobavaisoflavone	Flavonoids	Inhibit the N-formyl-L-methionyl-L-leucyl-L-phenylalanine (fMLP)-induced O_2_^–^ generation and/or elastase release	Chen et al. [180]

Quercetin	Isolated compound	Tea, fruits and vegetables	2-(3,4-Dihydroxyphenyl)-3,5,7-trihydroxy-4H-chromen-4-one	Flavonoid	Inhibits LOX and PDE4. Reduce leukotrienes and histamine release with a decrease in the IL-4 level. Inhibit prostaglandins release and the human mast cell activation by Ca_2_^+^ influx	Townsend et al. [181]; Mlcek et al. [182]

*Radix Rehmanniae Preparata*	Extract	Not reported	Catalpol	Glycoside	Inhibit IgE secretion. Decrease IL-4 and IL-5. Inhibit eosinophil infiltration and suppress eotaxin and its receptor CCR3. Reduce IL-5R*α* levels	Chen et al. [183]

Resveratrol	Isolated compound	Skin and barks of red fruits	Resveratrol (3,4,5-trihydroxystilbene)	Polyphenol	Decreases eosinophilia. Reduce neutrophil migration and inhibit PGD-2 release. Decrease IL-4 and IL-5 and also the hyperresponsiveness and mucus production	Lee et al. [184]; Hu et al. [185]; Chen et al. [186]

*Schisandra chinensis*	Extract	Dried fuits	*α*-Cubebenoate	Not reported	Suppress bronchiolar structural changes. Inhibit the accumulation of lymphocytes, eosinophils, and macrophages in BALF. Suppress IL-4, IL-13, and TGF-*β*1. Increase the intracellular Ca_2_^+^	Lee et al. [187]

Sea cucumber (*Holothurians*)	Tonic	Marine animal (Sea cucumber)	Holothurin A3, pervicoside A, and fuscocinerosides A	Toxins	Reduce COX enzymatic activity	Guo et al. [188]
*Selaginella uncinata* (Desv.)	Extract	Dried herbs	Amentoflavone, hinokiflavone, and isocryptomerin	Flavonoids	Attenuate hyperresponsiveness and goblet cell hyperplasia. Decrease IL-4, IL-5, IL-13, and IgE levels in serum. Upregulation of T2R10 gene expression and downregulation of IP3R1 and Orai1 gene expression. Suppression of eotaxin, NFAT1, and c-Myc protein expression	Yu et al. [189]

*Selaginella pulvinata*	Isolated compound	Air-dried powder of the whole plant of S. pulvinata	Selaginpulvilin A, selaginpulvilin B, and selaginpulvilin C	Phenol	Inhibit the PDE4	Liu et al. [190]

*Sideritis scardica*	Extract	Leaves	Echinacoside, verbascoside, luteolin, apigenin, caffeic acid, vanillic acid	Glycosides, flavonoids, and phenolic acids	Not elucidated	Todorova and Trendafilova [191]

*Siegesbeckia glabrescens*	Extract	Aerial roots	3,40-O-Dimethylquercetin, 3,7-O-dimethylquercetin, 3-O-methylquercetin, and 3,7,40-O-trimethylquercetin	Flavonoids	Reduce inflammatory cell infiltration in BALF. Decrease IL-4, IL-5, IL-13, eotaxin, and IgE. Reduce airway inflammation and mucus overproduction. Decrease iNOS and COX-2 expression and reduce NO levels	Jeon et al. [192]

Sitostanol	Isolated compound	Marketable synthetic compound	Sitostanol	Steroid	Suppresses IL-4 and IL-13 release	Brüll et al. [193]

Soft coral	Isolated compound	Sarcophyton ehrenbergi	Not reported	Prostaglandins	Inhibits PDE4	Cheng et al. [194]

Solanum *paniculatum* L	Extract	Fruits	Stigmasterol and *β*-sitosterol	Steroid	Reduce IL-4 and NO levels. Decrease IFN-*γ* without changes in IL-10 levels. Reduce NF-*κ*B, TBET, and GATA3 gene expression	Rios et al. [195]

Squill (*Drimia maritima* (L.) stearn) oxymel	Crude extract	Not reported	Scillaren A, scillirubroside, scilliroside, scillarenin, and proscillaridin A	Glycosides	Not elucidated	Nejatbakhsh et al. [196]

*Sorbus commixta Hedl.* (Rosaceae)	Methanolic extract	Fruits	Neosakuranin	Glycosides	Not elucidated	Bhatt et al. [197]

*Thuja orientalis*	Extract	Fruits	Cupressuflavone, amentoflavone, robustaflavone, afzelin, (+)-catechin, quercetin, hypolaetin 7-O-*β*-xylopyranoside, isoquercitrin, and myricitrin	Flavonoids	Reduce nitric oxide production and reduce the relative mRNA expression levels of inducible nitric oxide synthase (iNOS), IL6, cyclooxygenase-2, MMP-9, TNF-*α* in vitro. Decrease the inflammatory cell counts in BALF. Reduce IL-4, IL-5, IL-13, eotaxin, and IgE levels and reduce the airway hyperresponsiveness, in vivo. Attenuate mucus hypersecretion	Shin et al. [198]

Tonggyu-tang	Extract	*Ledebouriella divaricata* Hiroe*, Angelica koreanum Kitagawa, Angelica tenuissima Nakai, Cimicifuga heracleifolia Kom., Pueraria thunbergiana Benth., Ligusticum wallichii var. officinale Yook., Atractylodes lancea DC., Thuja orientalisl., Ephedra sinica Stapf., Zanthoxylum schinifolium S.Z., Asarum sieboldii var. seoulense Nakai, Glycyrrhiza glabra, Astragalus membranaceus var. mongholicus Bung, Xanthium strumarium* L.*, Magnolia denudate Desr., Mentha arvensis var. piperascens Makinv*	Not reported	Plant	Inhibit inflammatory cytokines (IL-4, IL-6, IL-8, and TNF-*α*). Suppress mitogen activated protein kinase (MAPK) and NF-*κ*B in mast cells and keratinocytes	Kim et al. [199]

*Trigonella foenum-graecum*	Extract	Seeds	Not reported	Flavonoids	Reduce IL-5, IL-6, IL-1*β*, and TNF-*α*. Reduce collagen deposition in goblet cells. Suppress inflammatory cells	Piao et al. [200]

*Tropidurus hispidus*	Oil	Fat of tropidurus hispidus	Croton oil, arachidonic acid, phenol, and capsaicin	Fatty acids and its derivated	Affect the arachidonic acid and their metabolites and reduce proinflammatory mediators	Santos et al. [201]

*Urtica dioica L*.	Extract	Leaves	Caffeic acid, gallic acid, quercetin, scopoletin, carotenoids, secoisolariciresinol, and anthocyanidins	Polyphenols, flavonoids, cumarin, and lignan	Reduce leucocytes and lymphocytes levels in serum. Inhibit the eosinophilia increase in BALF. Suppress inflammatory cells recruitment and attenuation of lipid peroxidation of lung tissues	Zemmouri et al. [202]

Verproside	Isolated compound	*Pseudolysimachion*	Verproside	Glycoside	Suppress the NF-*κ*B and TNF-*α* expression	Lee et al. [203]

Vitamin D	Isolated compound	Not reported	Calcitriol	Vitamin	Inhibit lymphocytes (Th1 and Th2) and reduces cytokines production	Szekely and Pataki [204]

Vitamin E	Isolated compound	Plant lipids	*α*-, *β*-, *γ*-, and *δ*-Tocopherols and the *α*-, *β*-, *γ*-, and *δ*-tocotrienols	Vitamin	Reduce airway hyperresponsiveness, IL-4, IL-5, IL-13, OVA-specific IgE, eotaxin, TGF-*β*, 12/15-LOX, lipid peroxidation, and lung nitric oxide metabolites	Cook-Mills and McCary [205]; Abdala-Valencia et al. [206]

*Vitex rotundifolia linn til* (Verbenaceae)	Methanolic extract	Fruits	1H, 8H-Pyrano [3, 4-c]pyran-1,8-dione	Not reported	Inhibit eotaxin, IL-8, IL-16, and VCAM-1 mRNA	Lee et al. [207]

*Viticis fructus*	Extract	Dried fruit	Pyranopyran-1,8-dione	Not reported	Inhibit eosinophils and lymphocytes cell infiltration into the BAL fluid. Reduce to normal levels of IL-4, IL-5, IL-13 and eotaxin. Suppress IgE levels	Park et al. [208]

Yu ping feng san	Extract	Radix Saposhnikoviae (Fangfeng), Radix Astragali (Huangqi), and Rhizoma Atractylodis macrocephalae (Baizhu)	Calycosin-7-O-*β*-d-glucoside, calycosin, formonetin, atractylenolide III, II, and I; 5-O-methylvisammioside, 8-methoxypsoralen and bergapten	Flavonoids, terpenoids, saponins, and furocoumarins	Inhibit TNF-*α*, IFN-*γ*, and IL-1*β*	Stefanie et al. [209]

*Zygophyllum simplex* L	Extract	Aerial parts	Isorhamnetin-3-O-*β*-D-rutinoside, myricitrin, luteolin-7- O-*β*-D-glucoside, isorhamnetin-3-O-*β*-D-glucoside, and isorhamnetin	Phenol	Inhibit NF-*κ*B, TNF-*α*, IL-1*β*, and IL-6	Abdallah and Esmat [210]

Ziziphus amole	Extract	Leaves, stems, barks, and roots	Alphitolic acid, sitosterol, ziziphus-lanostan-18-oico acid	Terpenoid and steroid	Inhibit myeloperoxidase activity	Romero-Castillo et al. [211]
